# An Assessment of Metallothionein–Cadmium Binding in Rat Uterus after Subchronic Exposure Using a Long–Term Observation Model

**DOI:** 10.3390/ijms232315154

**Published:** 2022-12-02

**Authors:** Marzenna Nasiadek, Joanna Stragierowicz, Anna Kilanowicz

**Affiliations:** Department of Toxicology, Medical University of Lodz, Muszynskiego 1, 90-151 Lodz, Poland

**Keywords:** cadmium, essential elements, metallothionein, rats, toxicity, uterus

## Abstract

Cadmium (Cd) is an environmental pollutant known to pose a public health issue. The mechanism of Cd toxicity on the uterus, including the protective role of metallothionein (MT), is still not fully understood. The aim of the study was to evaluate the degree of MT-Cd binding in the uterus of rats exposed *per os* to Cd at daily doses of 0.09, 0.9, 1.8 and 4.5 mg Cd/kg b.w. for 90 days. To assess the permanence of the bond, the rats were observed over long observation periods: 90 and 180 days after termination of exposure. Additionally, uterine concentration of Zn, Cu, Ca, Mg was determined. Cd leads immediately after exposure to a max. 30-fold increase in the concentration of Cd in the uterus, with only small amounts being bound to MT. After 90 days following termination of exposure, and especially after 180 days, an increase in MT-Cd concentration was noted for the three highest doses; even so, the degree of Cd binding by MT was still small. Additionally, the accumulation of Cd in the uterus disturbs the homeostasis of determined essential elements, manifested by a significant increase in Cu concentration and a decrease in Zn, Mg and Ca, especially 180 days after termination of exposure. The obtained results indicate that MT has only a slight protective role in the uterus and that Cd ions may have harmful effects not related to MT: directly on the uterine tissue, and indirectly by disturbing the homeostasis of its essential elements.

## 1. Introduction

Cadmium (Cd) is a widespread environmental pollutant from agricultural and industrial sources. Numerous epidemiological and cohort studies indicate that even environmental exposure to low Cd concentrations leads to a higher risk of damage to the kidneys, liver and skeletal system, as well as an increased occurrence of cardiovascular disorders, and even cancer [1,2]. Additionally, Cd has been included as an endocrine disruptor chemical (EDC), mainly due to the risk of adverse effects on the reproductive system. Epidemiological studies increasingly indicate a relationship between environmental exposure to Cd and an increased risk of hormone-dependent cancers in the reproductive organs of both women (endometrial and ovarian cancer, myomas) and men (prostate cancer) [3,4,5]. However, Cd has only been found to act as a carcinogen in the kidneys and lungs at occupational exposure [6]. While the effect of Cd on the male reproductive system has been well documented in both human and animal studies [7,8], its toxic effects on the functioning of the female reproductive system remain unclear. Our previous studies indicate that Cd accumulates in the uterus of women, and experimental animal studies have confirmed that it can accumulate for long periods of up to 180 days after the end of exposure [9,10,11]. The ability of Cd to accumulate not only in the kidney and liver but also in the uterus, ovaries and testes, and to exert toxic effects through oxidative stress, has also been demonstrated in human and animal studies [12,13,14,15]. Further research indicates that oral Cd exposure may lead to reduced fertility in female rats [12,16,17], significant disorders of the estrus cycle [16,17,18], changes in the histopathology of ovaries and uterus [10,11,12,13,19] and disturbed homeostasis of sex hormones [11].

In the general population, the main routes of exposure to Cd are contaminated food and inhalation of cigarette smoke [20,21]. Those with Fe deficiency (anemia, pregnant women and women of childbearing age) are more predisposed to Cd absorption [22,23]. Iron deficiency has also been associated with higher blood and urine Cd levels. Women, particularly those who are pregnant or of childbearing age, are at particular risk of exposure, as numerous studies have shown twice as much Cd absorption from food in women (10%) as in men (5%). Many studies indicate that Cd may be an etiological factor for abnormalities in the female reproductive system, such as the alteration of steroidogenesis pathway, menstrual cycle and reproductive hormone disorders, delay in puberty and menarche, loss of pregnancy, premature birth and reduced birth weight [18,24]. Therefore, the female reproductive organs (mainly the uterus and ovaries) are considered to be particularly sensitive to the toxic effects of Cd.

Metallothionein (MT) plays a key role in protecting against the harmful effects of Cd [25]. It is low-molecular-weight, cysteine-rich protein, whose genes are transcriptionally activated by metal ions (Cd, Cu, Zn, Ag, Hg, etc.) in biological systems [26]. This protein performs several functions, including cellular storage and/or transport of Zn and Cu ions to metalloenzymes and other metalloproteins; the degradation in cytoplasm of MT-Zn and MT-Cu complexes allows the transport of these metals to metalloenzymes. The liberation of Zn and Cu is connected with oxidative status, indicated by GSH/GSSG ratio and the presence of ROS (reactive oxygen species) and RNS (reactive nitrogen species) [27]. MT is also able to detoxification of metals (Cd, Hg, Pb, Ag, Co, Cu, Ni) [27] and scavenge free radicals produced in oxidative stress (also due to Cu-Zn superoxide dismutase). To date, four isoforms of MT (MT-1–MT-4) have been identified. Cd strongly induces the synthesis of isoforms MT-1 and MT-2, particularly in the case of acute and chronic Cd toxicity in the liver and kidneys; indeed, many studies have found cellular damage and/or liver and kidney dysfunction caused by heavy metals to be ameliorated by binding to MT. Nakamura et al. [28] also report the expression of three MT isoforms (MT-1, MT-2 and MT-3) in rat uterus. However, the mechanism by which MT may protect against Cd toxicity in the uterus and reproductive system remains unclear, and only a single study has described the uterine MT concentrations associated with Cd [28]. In addition, no studies exist on the concentration of Cd and its potential to remain bound to MT in the long-term following the end of exposure.

The degree of MT synthesis is also influenced by the concentration of Zn and Cu in tissues and in the diet [18]. In addition, Cd is known to interact with essential elements, such as Zn, Cu, Ca and Mg [29,30]. Although this data was obtained by examination of the critical organs for Cd (liver and kidney), none of the studies were performed on the uterus. An understanding of the dynamics of Cd in the uterus is of growing importance, as it has been suggested that such disturbances may be a key factor causing unexplained infertility or an abnormal course of pregnancy [31,32]. So far, Ca and Mg have been shown to play significant roles in regulating the excitability and contractility of the uterus [33,34], while disturbances in Zn and Cu homeostasis may lead to the induction of oxidative stress and, consequently, to cell apoptosis in this organ [35,36].

In conclusion, the mechanism of toxic action of Cd in the uterus, and many other tissues, is multidirectional and mainly involves the induction of ROS, which has already been well studied [15,20], as well as interaction with essential metals, which has mainly been proven so far in the liver and kidneys [29,30]; however, no such data exist for the uterus. Due to the important protective role played by MT against toxic metals, including Cd, and its ability to transport essential metals in other organs, and the lack of information in this topic in current literature, there is a clear need to investigate its role in the uterus following exposure to Cd.

Therefore, the aim of this study was to investigate the effect of subchronic (90-day) *per os* administration of Cd in four different doses (in the range: 0.09–4.5 mg Cd/kg b.w.) on its concentration and degree of binding to MT in the rat uterus. Additionally, the study also assesses the effect of Cd administration on the homeostasis of essential elements (Zn, Cu, Ca and Mg) in the rat uterus. Unlike previous research, the present study uses long observation periods, i.e., 90 and 180 days after the termination of exposure, to assess the permanence of the values taken immediately after Cd administration.

## 2. Results

**Figure 1:**
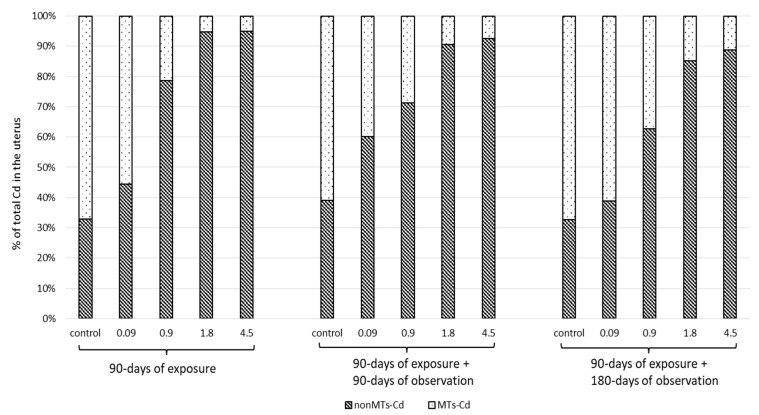
Cd bound to MT (MT-Cd) and Cd not bound to MT (nonMT-Cd) in the % pool of T-Cd in the uterus following 90-day oral exposure to CdCl_2_, and at 90 days and 180 days post-exposure.

**Figure 2:**
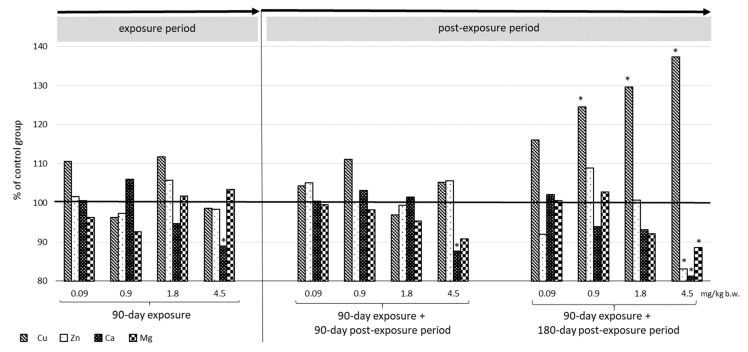
Changes in the concentrations of selected trace elements (Cu, Zn, Ca and Mg) in rat uterus given in % in relation to the control group (marked line 100%) after 90-day oral exposure to Cd, and after 90 and 180 days post-exposure; *p* ≤ 0.05 (*—vs. control group).

The results obtained immediately after repeated (90 days) administration of CdCl_2_ and after the subsequent 90- and 180-day observation periods are summarized in Table 1 and Figure 1 and Figure 2. No changes in animal behavior or appearance were noted throughout the whole experiment, i.e., the 90-day administration time or the additional 90-day or 180-day observation periods; all rats survived until the end of the study. No significant changes in body weight were noted (Figures S1–S3) nor in feed and water intake during all used periods (Figures S4–S9). Changes in relative uterus weight are presented in Figure S10.

### 2.1. Cd and MT

As shown in Table 1 and Figure 1, administration of CdCl_2_ caused significant changes in the levels of total Cd (T-Cd), Cd bound with metallothionein (MT-Cd) and Cd not bound to metallothionein (nonMT-Cd). Almost all Cd doses (0.9–4.5 mg/kg b.w.) were associated with significantly elevated levels of all determined biomarkers in rat uterus. The observed effects were dose dependent. Exposure to 0.9 mg Cd/kg b.w. was found to result in high levels of T-Cd being maintained (about five times higher compared to the control group) for 90 days (153.97 ± 31.16 ng/g vs. 28.03 ± 6.81 ng/g) and even 180 days (148.68 ± 28.33 ng/g vs. 30.34 ± 1.89 ng/g) after the end of the exposure. Only at higher exposure doses (1.8 and 4.5 mg/kg b.w.) was a slight reduction in T-Cd observed after 90 (487.57 ± 50.92 and 945.78 ± 16.83 ng/g) and 180 (452.64 ± 95.26 and 896.23 ± 21.33 ng/g) days of observation compared to the concentrations immediately after the end of the exposure (685.63 ± 35.90 and 1021.6 ± 76.36 ng/g) (Table 1). 

The distribution of MT-Cd and nonMT-Cd in the T-Cd pool is presented in Table 1 (given values in ng/g) and in Figure 1 (%). These data show that only the lowest dose of Cd (0.09 mg/kg b.w.) is associated with no significant changes in T-Cd, MT-Cd and nonMT-Cd concentrations immediately after the end of exposure and 180 days after exposure compared to the corresponding amounts obtained in control group. For all other concentrations and sample points, a greater level of nonMT-Cd was observed in the T-Cd pool, and its % increased with the applied dose. The ratio of MT-Cd to nonMT-Cd changed with the length of observation, which was especially visible at doses of 0.9 mg/kg b.w. and higher. For these doses, the participation of MT-Cd in the T-Cd pool increased over time, and this was especially marked for the dose of 0.9 mg/kg b.w.; this is evidenced by the observed increase in MT-Cd concentrations over time: 32.28 ± 4.46 ng/g (22%) immediately after exposure; 44.31 ± 3.03 ng/g (29%) after 90 days of observation and 55.44 ± 5.98 ng/g (37%) after 180 days (Table 1 and Figure 1). For the remaining exposure levels (1.8 and 4.5 mg/kg b.w.), MT-Cd concentrations increased significantly and more markedly over time; however, they did not represent such a significant % change in the T-Cd pool when combined with nonMT-Cd.

### 2.2. Determination of Trace Elements (Cu, Zn, Ca and Mg) in Uterus Tissue

The determinations of selected trace elements in the rat uterus are presented in Figure 2. The findings indicate that exposure to Cd leads to significant changes in the concentrations of some elements, especially after exposure to the highest dose (4.5 mg/kg b.w.). The concentration of Ca significantly decreased immediately after exposure to the highest dose of Cd; however, no such reduction was observed for the remaining trace elements (Cu, Mg and Zn). After 90 days of observation, all analyzed trace elements apart from Ca demonstrated slight changes at the highest Cd dose. After 180 days, all analyzed trace elements demonstrated large changes at the highest dose of Cd (4.5 mg/kg b.w.). Among these, the most notable changes were observed for Cu, which demonstrated a significant increase after exposure to Cd at 0.9 and 1.8 mg/kg b.w. A significant decrease in Ca, Zn and Mg concentrations were observed at the highest dose (4.5 mg/kg b.w.), with the reduction being dependent on the level of Cd.

## 3. Discussion

Cd is a ubiquitous metal in the environment, and the general population is subject to constant exposure, mainly through the diet and additionally through smoking [20,21]. Moreover, following its qualification as an EDC and its proven high toxicity, it has been recognized as one of the most dangerous environmental pollutants [37]. Increasing numbers of studies indicate that environmental exposure to this metal may be one of the factors involved in the etiology of disorders associated with the female reproductive organs, including infertility, polycystic ovary syndrome, premature puberty [38,39,40,41] or an increased risk of hormone-dependent tumors in the uterus [3,4,42,43]. Indeed, numerous animal studies have provided convincing evidence about the significant adverse effects of Cd on the female reproductive organs [10,11,12,16,34,44,45]. However, the mechanism of the uterine toxicity of Cd is still poorly understood.

The protective role of MT in Cd toxicity in different organs has so far been well documented in the kidney and liver [26,27,46]. The most important property of MT is their characteristic amino acid composition, with a high content of cysteine (and thus sulfur) [26,47], which enables them to bind various metals, such as Cd, and reduce their toxicity. It has been demonstrated in numerous studies that Cd ions not bound to MT are responsible for the toxic effects of Cd in the liver and kidneys [26,27,46,48]. This indicates that as in other organs, possibly including the uterus, MT may be involved in Cd accumulation and toxicity. This theory is confirmed by studies describing the increase in the expression of MT-1–MT-3 genes in the uterus, of which only MT-1 and MT-2 play an important role in the accumulation of Cd in this organ [28]. 

Therefore, the present study assesses the extent to which Cd binds to MT in the uterus of rats exposed sub-chronically to Cd at four different doses. To assess the stability of the bond, a long observation period of up to 180 days after the end of exposure was used. The two lowest doses (0.09 and 0.9 mg/kg b.w.) closely corresponded with the level of environmental exposure: the range of Cd concentrations in the uterus of rats for these doses (37.48–153.97 ng/g wet tissue) were similar to the concentration levels in women environmentally exposed to Cd (10–150 ng/g wet tissue) [9,14]. 

Our findings indicate that after the end of the 90-day exposure, Cd binds to MT in the uterus depending on the dose, reaching the highest value after a dose of 4.5 mg/kg b.w. (50.76 ± 2.99 ng/g wet tissue). Binding of Cd to MT in the uterus, expressed in %, was most effective only after the administration of the lowest dose (0.09 mg/kg b.w. = 55%), while this trend was inversely proportional to Cd dose, and was approximately 5% for the two highest doses. This effect may account for the fact that the toxicity of Cd in the uterus, expressed as reactive oxygen species (ROS) induction, was found to increase with the dose (based on the same Cd dosing model and observation time) [11]. 

A literature review shows that only Nakamura et al. [28] has conducted a similar study of the degree of Cd binding by MT in rat uterine tissue after subchronic exposure to Cd in a similar dose range (1–5 mg/kg b.w.); their findings also demonstrate a low degree of binding for the highest doses, i.e., 10%. However, the authors did not investigate lower doses, which better reflect the true environmental exposure by women (the present study included a dose of 0.09 mg/kg b.w. for this purpose).

Our study presents the first assessment of MT-Cd concentration and its bound percentage in uterine tissue over a long-term observation period of 90 and 180 days following a 90-day dosing period. Interestingly, the concentration of MT-Cd was found to increase over the post-exposure 90- and 180-day observation periods, especially for the higher doses of Cd (0.9–4.5 mg/kg b.w.). However, while an approximately twofold increase in MT-Cd concentration (67.45 ± 1.39 and 101.02 ± 17.81 ng/g wet tissue, respectively) was noted for the two highest doses (1.8 and 4.5 mg/kg b.w.) after 180 days of observation compared to the end of exposure (35.90 ± 10.25 and 50.76 ± 2.99 ng/g wet tissue, respectively), the amount of Cd bound to MT remained low, amounting to approximately 15% and 11% for the respective doses. 

In the case of the lowest dose (0.09 mg/kg bw.), the percentage of Cd binding to MT decreased only after 90 days of observation while the nonMT-Cd content increased; however, these changes seemed to normalize after the 180 days of observation, probably due to increased MT synthesis. It is possible that, like in other tissues, the Cd which was bound to other proteins containing -SH groups may dissociate and bind to the de novo synthesized MT. This may explain why Cd has such a long biological half-life in the body [46], as would also be the case in the uterus.

The contribution of the MT defense mechanism in kidney and liver seems to be more pronounced than in uterus. A previous study found the proportion of MT-Cd to T-Cd to be 66% (kidney) and 86% (liver), after subchronic exposure with 0.6 mg Cd/kg b.w. for eight weeks [49]. In comparison, in the present study, a similar dose of Cd (0.9 mg/kg b.w.) in the uterus resulted in an approximate three- or four-fold lower score (22%) than in the kidneys or liver, respectively.

It is difficult to compare our findings with others, as most studies on other tissues only assess the general pool of MT concentration without assessing the degree of MT-Cd [48,50,51]. Only slight MT induction has been noted in the female and male reproductive organs in vitro and in vivo, with a reduced protective role: no significant increase in either mRNA synthesis or MT concentration was observed after administration of Cd [52]. In contrast, Dalton et al. [53] found the male reproductive organs to demonstrate greater sensitivity to the effects of Cd, indicating that even MT-1 overexpression in mice did not protect against testicular necrosis induced by Cd toxicity. It is possible that this sensitivity of the reproductive organs is due not only to the low contribution of MT, but also to other mechanisms of nonMT-related Cd toxicity.

Since the mechanism of action of Cd toxicity is based to some extent on an interaction with trace elements, such as Zn, Cu, Ca and Mg, we also examined whether Cd treatment disturbs their homeostasis in the uterus. So far, this has been better documented in the liver, kidney, spleen, gut, and bone tissue [51,54,55]. Indeed, the influence of Cd on the metabolism of these essential elements in the uterus, depending on Cd dose and time after exposure, appears unresearched. MT may also play a role in the pathomechanism of these disorders, because this protein is known to play roles in maintaining homeostasis and transporting essential metals, i.e., Zn and Cu [27,56,57].

Our present findings confirm disturbances in the pool of the examined elements (Zn, Cu, Ca and Mg) in the uterus of rats, and although no significant changes in the homeostasis of these elements were found at the end of the 90-day Cd treatment period, except for Ca, more significant changes were found after a long (180-day) follow-up period. After 180 days of observation, following the end of exposure, a significant decrease in Zn, Ca, Mg and an increase in Cu concentration was noted at the highest dose (4.5 mg/kg b.w.); these changes were also observed for Cu at the lower dose, i.e., similar to environmental exposure (0.9 mg/kg b.w.), and at 1.8 mg/kg b.w. While many authors note changes in the levels of essential elements directly after the end of subchronic experiment, none used such a long observation period as used in our study [30,51,58,59].

The interactions between Zn and Cd result from their similar chemical properties and common absorption mechanisms, and mainly from the shared ability of these metals to induce MT synthesis and bind to them [35]. Reducing the concentration of Zn in the tissue may have an adverse effect, as it is associated with inter alia indirect regulation of oxidant production, leading to an increase in ROS [60]. In addition, increased cellular apoptosis may arise as a consequence of the intracellular decrease in Zn concentration [61]. In vitro studies have shown that Zn inhibits many proteins associated with apoptosis, including Ca and Mg-dependent caspases or proteases [61]. Persistent ROS induction has previously been noted in rat uterine tissue after exposure to the same doses of Cd and exposure times [11], and it cannot be ruled out that this may also result from a decrease in Zn concentration.

Our findings also indicate that uterine Ca concentration only decreased after administration of the highest dose of Cd; however, this decrease lasted from the end of exposure for the entire 180 days observation period. Cd is known to inhibit the absorption of Ca, specifically calcium channels, causing a reduction in their flow. A high concentration of Ca^2+^ ions is an essential component of the uterine contraction pathway, which ensures the activity of myosin light chain kinase and myosin phosphorylation. An element closely related to Ca homeostasis is also Mg. In our study, we showed a statistically significant decrease in Mg concentration in the uterus, most markedly after 180 days post-exposure. Mg activates ATP, influencing the transport of Ca to intracellular spaces, which may reduce the concentration of Ca^2+^ ions in uterine cells. Since Ca^2+^ and Mg^2+^ ions regulate the excitability and contractility of the uterus, the simultaneous reduction of Ca and Mg concentrations in this organ may lead to a decrease in uterine muscle contractility, which may have a significant negative impact, especially during childbirth [33,34].

As in the uterus, changes in Ca concentration have been demonstrated in bones during chronic exposure to Cd [30,62]. Cd is proposed to influence bone metabolism via two mechanisms of action, leading to a decrease in bone density [63]. The first mechanism involves direct action on bone cells [64], while the second works indirectly by increasing urinary Ca and P excretion, thus inducing renal failure, reducing vitamin D synthesis and reducing Ca absorption in the gastrointestinal tract [63,65]. The latter indirect mechanism may also be responsible for the reduction in Ca concentration observed in the uterine tissue at all tested time points.

Another important essential metal whose homeostasis was disturbed by Cd in the uterus was Cu. Interestingly, Cu demonstrated an opposite effect to the other essential elements: a significant increase in concentration was noted after administration of 0.9–4.5 mg/kg b.w. Cd but only after the longest follow-up period, i.e., 180 days. Similar increases in Cu concentration have also been observed in the liver and kidneys of rats after chronic oral exposure to Cd [29,30]. Like Zn, Cu is an essential trace element involved in a variety of biological functions, its homeostasis is controlled by proteins such as MT and membrane transporters, and is also a cofactor of many enzymes that protect the cell from oxidative stress. In addition, in the presence of reducing agents, Cu is capable of forming hydroxyl radicals in the Haber–Weiss reaction [66], which indicates that disturbances in Cu concentrations may play a significant role in the induction of oxidative stress in this organ. Numerous studies indicate that both the concentration of Cu in the serum and in the neoplastic tissues of the uterus and other organs is increased following Cd environmental exposure [67,68], and a positive correlation has been shown between the concentration of Cu and cancer progression [69]. Therefore, it cannot be ruled out that long-term Cd accumulation as a result of increased Cu concentration may contribute to the etiology of neoplasms in the uterus. Indeed, several recent studies have indicated a correlation between dietary Cd intake and increased risk of endometrial cancer [3,4].

## 4. Materials and Methods

### 4.1. Chemicals

Cadmium chloride (CdCl_2_ × 2.5 H_2_O) was purchased from Sigma-Aldrich (St. Louis, MO, USA). Trace-pure 65% nitric acid (J.T. Baker, Ultrex II Analyzed, Phillipsburg, NJ, USA) and Astasol-Mix (Analytical Ltd., Prague, Czech Republic) were applied as stock solutions of Zn, Cu, Ca, Mg and Cd assigned for atomic absorption spectrometry (AAS method). The solution of lanthanum was used as a matrix modifier in Ca and Mg analysis. The analytical quality of the metals and trace elements was confirmed using the Standard Reference Material Bovine Liver (No. 1577b; National Institute of Standards and Technology, Gaithersburg, MD, USA). Demineralized ultrapure Milli-Q plus water, reagent grade (Millipore, Merck KGaA, Darmstadt, Germany) was used in all measurements. 

### 4.2. Experimental Protocol

All procedures conducted on the rats were approved by the Local Animal Ethical Committee of the Medical University of Lodz (LKE 46/LB/481/2009; 14/LB481/DLZ/2012).

The study was carried out in a population of 120 adult female Wistar rats (9–10 weeks old), with a mean initial body weight of 207 ± 20 g; all had completed at least three consecutive regular estrous cycles. The animals were kept in polypropylene cages with free access to tap water and a diet low in phytoestrogen content (Ssniff R/M-H, Ssniff Spezialdiäten GmbH, Soest, Germany), under standard laboratory indoor conditions: a 12 h light/dark cycle, controlled temperature of 22 ± 1 °C, relative humidity of 50–60%. The rats were allowed to acclimate for two weeks prior to use in the study. To identify regular estrous cycles, cytological analyses of vaginal smears by light microscopy were performed.

The experimental model has been described in detail previously [11]. Following acclimatization, the rats were randomly allocated into three groups (each *n* = 40): A, B and C, which were then divided into five subgroups (each *n* = 8). The experimental design and established dose are presented on Table 2. The Cd subgroups (from each group: A, B, and C) received Cd as aqueous solution of Cd chloride by oral gavage for 90 days. The following doses were applied: 0.09, 0.9, 1.8 and 4.5 mg Cd/kg b.w. per day. These correspond to subchronic exposure. Doses of Cd were selected to represent environmental levels of exposure in the general population based upon literature data [70]. After 90 days of exposure, group A was sacrificed, while group B was subjected to a 90-day observation period (three months) and C to a 180 day period (six months). The rats from the pure control subgroups (from groups A–C) were administered distilled water following the same protocol used for the rats exposed to Cd. The daily feeding habits, water intake, body weight, and gross behavioral changes of all the animals were carefully observed throughout the whole experimental periods. 

#### Euthanasia, Tissue Collection, Preservation and Mineralization

Female rats which were found to be in estrus were weighed and underwent euthanasia after 90 days of exposure (Group A), and then after 90 days following exposure (Group B) and 180 days after exposure (Group C). 

The uteri were dissected out, trimmed of fat, weighed and divided into two parts: the first was used for trace element analysis and second for MT. Every part of the uterine tissue was stored in cryo-tubes at −80 °C until analysis. All tubes had been washed properly within 24 h in 10% nitric acid.

### 4.3. Cd, Zn, Mg, Ca, Cu Assessment

Cd and Cu concentrations were analyzed in uterus tissue, using a Hitachi Z-8270 GF-AAS flameless atomic spectrometer (Hitachi, Ltd., Tokyo, Japan) with Zeeman-type background correction, autosampler and pyrocoated tube. Zn, Ca and Mg were determined using flame atomic absorption spectrometry on F-AAS (Avanta GM; GBC Scientific Equipment Pty Ltd., Melbourne, Australia). Tissue sample digestion was performed with a MarsXpress microwave digestion system (CEM Corporation, Matthews, NC, USA) with nitric acid. For determination of Cd and trace elements concentrations, the samples were prepared in duplicate. To assess Cd, Cu, Mg and Ca, mineralized tissues were additionally diluted (two to eight times) in relation to the measured metal. For each sample, three parallel independent determinations were made and the mean values were accepted. The concentrations of Cd and Cu, Zn, Ca, Mg in the tissues were reported as ng/g wet tissue and µg/g wet tissue. The accuracy of these measurements was confirmed against reference material (Bovine Liver 1577b). For 96% of the determinations, the repeatability error did not exceed 10%. The precision of the measurements, expressed as a coefficient of variation for Cd, Cu, Zn, Mg and Ca in the range of the samples analyzed in this study was <5.6%, <3.3%, <9.4%, <4.8% and <9.5%, respectively. 

### 4.4. MT Determinations 

The parts of the uterus were homogenized well in TRIS-HCl to 10% homogenates using T25 Kika labotechik homogenizer, Bandelin Sonplus HD 2070. The level of MT was determined using a modified cadmium-hemoglobin affinity assay according to Onosaka et al. [71] and Eaton and Toal [72], with the final Cd measurement performed by graphite furnace atomic absorption spectrometry with Zeeman correction (Hitachi Z-8270). The detection limit for the Cd was 0.1 ng/g wet tissue. 

### 4.5. Statistical Analysis

All data were analyzed in STATISTICA, version 10.1. (StatSoft Polska Sp. z o.o., Kraków, Poland). The Kruskal–Wallis one way analysis of variance was used, followed by a pair-wise comparison of selected means with Levene’s test. The statistical significance was set at *p* ≤ 0.05.

## 5. Conclusions

The study we conducted showed that although Cd accumulates in the uterus in a dose-dependent manner, both immediately after the exposure and in a long-term observation period, it is bound to MT to only an insignificant degree. This suggests that the detoxication of Cd by MT is not sufficient, while Cd ions, not bound to MT, may have a toxic effect in this organ. The study also proved that Cd disturbs the homeostasis of essential elements in the uterus, especially Cu. Taking into consideration the whole life accumulation of Cd and its multidirectional toxicity on the uterus, it suggests the conclusion that Cd environmental pollution may impair reproduction in many species, including human women.

## Figures and Tables

**Table 1:** Cd bound to MT (MT-Cd) and Cd not bound to MT (nonMT-Cd) in the total Cd pool (T-Cd) in the uterus following 90-day oral exposure to Cd, and 90 and 180 days post-exposure.

Treatment	Dose [mg/kg b.w.]	T-Cd [ng/g]	MT-Cd [ng/g]	nonMT-Cd [ng/g]
Group A (90-Day Exposure)				
Pure control	0	29.24 ±10.11	19.65 ± 1.91	9.59 ± 1.02
Cd	0.09	37.48 ±12.41	20.60 ± 9.49	16.48 ± 6.94
Cd	0.9	145.82 ± 11.41 ^a^	32.28 ± 4.46 ^a^	118.81 ± 6.88 ^a^
Cd	1.8	685.63 ± 35.90 ^a^	35.90 ± 10.25 ^a^	645.23 ± 35.40 ^a^
Cd	4.5	1021.6 ± 76.36 ^a^	50.76 ± 2.99 ^a^	968.20 ± 75.99 ^a^
Group B (90-day exposure and 90-day post-exposure period)				
Pure control	0	28.03 ± 6.81	16.94 ± 4.32	10.89 ± 2.01
Cd	0.09	48.64 ± 10.21 ^a^	19.38 ± 4.79 ^a^	29.21 ± 6.31 ^a^
Cd	0.9	153.97 ± 31.16 ^a^	44.31 ± 3.03 ^a^	109.97 ± 30.18 ^a^
Cd	1.8	487.57 ± 50.92 ^a^	45.51 ± 3.48 ^a^	439.98 ± 50.06 ^a^
Cd	4.5	945.78 ± 16.83 ^a^	69.47 ± 6.69 ^a^	875.53 ± 15.43 ^a^
Group C (90-day exposure and 180-day post-exposure period)				
Pure control	0	30.34 ± 1.89	20.45 ± 1.71	9.89 ± 1.12
Cd	0.09	38.95 ± 13.09	23.84 ± 9.76	15.18 ± 12.98
Cd	0.9	148.68 ± 28.33 ^a^	55.44 ± 5.98 ^a^	93.32 ± 28.01 ^a^
Cd	1.8	452.64 ± 95.26 ^a^	67.45 ± 1.39 ^a^	385.49 ± 94.83 ^a^
Cd	4.5	896.23 ± 21.33 ^a^	101.02 ± 17.81 ^a^	795.62 ± 20.14 ^a^

All values are expressed as mean (n = 8) ± SD. ^a^ *p* ≤ 0.05, significantly different from control group.

**Table 2:** Experimental groups, periods, dose and tested parameters.

Group (*n* = 40)	Period	Subgroup (*n* = 8)	Dose (mg/kg b.w.)	Determinations in Uterus
A	90-day of exposure	Control	water	T-Cd, Cd-MT and nonCd-MT (calculated) Essential elements: Zn, Cu, Ca, Mg
		Cd	0.09	
		Cd	0.9	
		Cd	1.8	
		Cd	4.5	
B	90-day of exposure + 90-day of observation	Control	water	T-Cd, Cd-MT and nonCd-MT (calculated) Essential elements: Zn, Cu, Ca, Mg
		Cd	0.09	
		Cd	0.9	
		Cd	1.8	
		Cd	4.5	
C	90-day of exposure + 180-day of observation	Control	water	T-Cd, Cd-MT and nonCd-MT (calculated) Essential elements: Zn, Cu, Ca, Mg
		Cd	0.09	
		Cd	0.9	
		Cd	1.8	
		Cd	4.5	

Abbreviations: T-Cd: total Cd concentration; Cd-MT: Cd bound with MT; nonCd-MT: non bound Cd with MT.

## Data Availability

Not applicable.

## Supplementary Materials

The supporting information can be downloaded at: https://www.mdpi.com/article/10.3390/ijms232315154/s1.
