# Supplementary material for: An Assessment of Metallothionein–Cadmium Binding in Rat Uterus after Subchronic Exposure Using a Long–Term Observation Model
Source: Int J Mol Sci. 2022 Dec 2;23(23):15154. doi: 10.3390/ijms232315154 (PMC9738218; doi:10.3390/ijms232315154)
Supplement: Supplementary file 1 [file ijms-23-15154-s001.zip › ijms-2052610-supplementary.docx]

An Assessment of Metallothionein–Cadmium Binding in Rat Uterus after Subchronic Exposure using a Long–Term Observation Model

Marzenna Nasiadek^*^, Joanna Stragierowicz, Anna Kilanowicz

Department of Toxicology, Medical University of Lodz, Muszynskiego 1, 90-151 Lodz, Poland

^*^ Correspondence: [marzenna.nasiadek@umed.lodz.pl](mailto:marzenna.nasiadek@umed.lodz.pl)

List of contents:

**Figure S1.** Growth curves for female rats exposed to Cd for 90 days. All values are expressed as mean (n=8) ± S.E.M.

**Figure S2.** Growth curves for female rats exposed to Cd for 90 days and 90 days of post-exposure period. All values are expressed as mean (n=8) ± S.E.M.

**Figure S3.** Growth curves for female rats exposed to Cd for 90 days and 180 days of post-exposure period. All values are expressed as mean (n=8) ± S.E.M.

**Figure S4.** Daily consumption of fodder on selected weeks for female rats exposed to Cd for 90 days. All values are expressed as mean (n=8).

**Figure S5.** Daily consumption of fodder on selected weeks for female rats exposed to Cd for 90 days and 90 days of post-exposure period. All values are expressed as mean (n=8).

**Figure S6.** Daily consumption of fodder on selected weeks for female rats exposed to Cd for 90 days and 90 days of post-exposure period. All values are expressed as mean (n=8).

**Figure S7.** Daily consumption of water on selected weeks for female rats exposed to Cd for 90 days. All values are expressed as mean (n=8).

**Figure S8.** Daily consumption of water on selected weeks for female rats exposed to Cd for 90 days and 90 days of post-exposure period. All values are expressed as mean (n=8).

**Figure S9.** Daily consumption of water on selected weeks for female rats exposed to Cd for 90 days and 90 days of post-exposure period. All values are expressed as mean (n=8).

**Figure S10.** Changes in relative uterus weight presented as % of final body weight. All values are expressed as mean (n=8) ± S.E.M.

Figure S1.

Figure S2.

Figure S3.

Figure S4.

Figure S5.

Figure S6.

Figure S7.

Figure S8.

Figure S9.

Figure S10.
